# Prevalence and influencing factors of perinatal depression across different stages of pregnancy and postpartum: a cross-sectional study in China

**DOI:** 10.3389/fpubh.2025.1692231

**Published:** 2026-01-05

**Authors:** Bing Fu, Siyuan Zhang, Linghui Xiang, Mengsi Cai, Mengxin Ou, Guanxiu Tang, Ruirui Huang, Jun Lei, Xin Lu

**Affiliations:** 1 Department of Obstetrics and Gynecology, The Third Xiangya Hospital of Central South University, Changsha, China; 2 College of Systems Engineering, National University of Defense Technology, Changsha, China; 3 School of Nursing, Hunan University of Medicine, Huaihua, China

**Keywords:** perinatal depression, prevalence, influencing factors, various pregnancy stages, cross-sectional study

## Abstract

**Background:**

Perinatal depression (PND) is a major public health concern, affecting both maternal and infant health. While numerous studies have examined risk factors for PND, most focused on single pregnancy stages, with limited evidence spanning the full perinatal period. This study aimed to assess the prevalence of PND across pregnancy and postpartum, and identify stage-specific influencing factors among Chinese women.

**Methods:**

A cross-sectional study was conducted among 1,047 participants in early pregnancy (T1: ≤ 13 weeks), 543 in mid-pregnancy (T2: 14–27 weeks), 421 in late pregnancy (T3: ≥ 28 weeks to delivery), and 424 within 6 weeks postpartum (T4), recruited from The Third Xiangya Hospital of Central South University. Demographic, psychological, and physiological data were collected, and PND was assessed using the Chinese version of the Edinburgh Postnatal Depression Scale (EPDS, cutoff ≥10).

**Results:**

The prevalence of PND was 30.95% at T1, 20.81% at T2, 18.29% at T3, and 28.07% at T4. Influencing factors varied across stages: in T1, relationship status, partner characteristics, family functioning, stress, and sleep quality were significant; in T2, health insurance, life events, social support, and sleep quality played key roles; in T3, family income, partner occupation, and family functioning were associated with PND; in T4, minority status, parental self-efficacy, history of ectopic pregnancy, and infant feeding method emerged as significant predictors.

**Conclusion:**

The prevalence and determinants of PND differ markedly across pregnancy and postpartum. These findings highlight the importance of stage-specific screening and tailored interventions: addressing relationship and family dynamics in early pregnancy, social support and healthcare access in mid-pregnancy, financial stability in late pregnancy, and parenting-related stressors postpartum. Targeted strategies may improve prevention and management of PND and ultimately enhance maternal mental health outcomes.

## 1 Introduction

Perinatal depression (PND) is a non-psychotic depressive episode, varying from mild to severe, that occurs during pregnancy (antenatal depression) and up to one year postpartum (postnatal depression) (1–6). PND has been shown to negatively impact both maternal and infant health (7–9). Physically, PND is associated with increased risks of spontaneous abortion (10), substance abuse (11), assisted delivery (e.g., cesarean section or instrumental delivery) (10, 12), and maternal suicide, which accounts for 20% of postpartum deaths (13). For infants, PND increases the likelihood of preterm birth (14), growth retardation (7, 10), and diarrheal infections (7, 10), adversely affecting both maternal and infant health. Psychologically, PND can disrupt maternal mental well-being and impair mother-infant interactions, leading to suboptimal mother-infant relationships (15), and potentially resulting in long-term negative effects on infant cognitive, socio-emotional, and interpersonal development (8, 15–18).

Research indicates that approximately one in five women experiences mental health challenges during the perinatal period, including depression, anxiety, and thoughts of suicide or self-harm (19–23). The perinatal period represents a particularly high-risk window for women's mental health, yet these issues have long been neglected (24). Depression has consistently remained at the forefront of public health research, as it is not only among the most common disorders but also one that is frequently underrecognized and undertreated (25). This lack of recognition has contributed to persistent shortcomings in care and has amplified its profound effect for maternal wellbeing, infant development, and family health (26, 27). The prevalence of PND is estimated to affect 10–15% of women in developed countries, with significantly higher rates observed in underdeveloped regions (1, 28). This disparity may be due to factors such as limited resources and inadequate healthcare systems. Consequently, research focused on the early detection and prevention of PND in developing countries is crucial, as it can reduce the social and economic burdens of PND on both families and society.

Previous studies have identified various factors associated with an elevated risk of PND (23, 29–33). These risk factors can be categorized into three broad groups: socio-demographic factors (e.g., lower maternal education, younger maternal age, poorer family economic status, and unfavorable marital status) (30, 33–35), psychological factors (e.g., adverse life events, lack of social support, personality traits, and life stress) (33–36), and physiological or biological factors (e.g., chronic illness or medical conditions) (30, 33, 35, 36). Pregnancy involves significant physiological changes, with each trimester potentially affecting the mental health of pregnant women differently (37, 38). Understanding the risk factors for PND across different trimesters provides a more comprehensive view of the challenges pregnant women face and enables timely interventions.

However, existing studies have largely focused on specific stages of pregnancy (9, 39), rather than examining stage-specific patterns across the perinatal period. This narrow focus limits our understanding of the temporal dynamics of PND vulnerability, which is especially critical given that physiological and psychological changes during pregnancy are not uniform but vary significantly across trimesters. Without a more comprehensive and stage-specific perspective, opportunities for timely screening and targeted interventions may be missed.

To address this gap, we conducted a cross-sectional study at The Third Xiangya Hospital of Central South University in Changsha, Hunan Province, China, aiming to identify stage-specific risk patterns of perinatal depression (PND) across four key periods: early pregnancy (T1), mid-pregnancy (T2), late pregnancy (T3), and the postpartum stage (T4). The Third Xiangya Hospital of Central South University, a well-known Grade-A tertiary hospital in China, holds recognized academic and clinical authority in obstetrics and maternal-child health. Situated in a provincial capital that integrates economically developed urban centers with resource-limited peri-urban areas, Changsha exemplifies the socioeconomic gradients characteristic of many low- and middle-income countries (LMICs), where rapid development often coincides with unequal access to healthcare. Our study design allows for a fine-grained temporal analysis that offers a cross-sectional snapshot of stage-specific differences, identifies vulnerable windows for intervention, and generates timely insights to inform tailored screening and prevention strategies for diverse groups of women in LMIC settings.

## 2 Methods

### 2.1 Study participants

This study was designed as a series of four independent cross-sectional surveys. At each of the four key stages, a distinct group of women attending routine obstetric visits at The Third Xiangya Hospital of Central South University between October 2022 and December 2023 was recruited to capture stage-specific risk patterns of perinatal depression in real-world clinical populations, as each sample reflects women who are actually present in care at that particular stage, thereby enhancing the representativeness of findings for each perinatal period. The Third Xiangya Hospital of Central South University is a large-scale, comprehensive, Grade III hospital with over 2000 beds and an annual patient volume exceeding 500,000 visits. As a leading institution in the region, it is spearheading the construction of the National Regional Medical Center for Obstetrics and Gynecology, further solidifying its role as a key healthcare provider in the area. With its high patient volume and well-established clinical pathways, we were able to implement standardized recruitment across different stages of pregnancy, yielding a study sample with broad demographic diversity. All participants provided written informed consent prior to participation. The study protocol was approved by the Ethics Committee of The Third Xiangya Hospital of Central South University (approval number: 2022–S270) and was conducted in accordance with the ethical standards of the Declaration of Helsinki.

### 2.2 Data collection

Data collection was conducted by a team of trained obstetricians and nurses, all of whom underwent specialized training to ensure accuracy and consistency. The structured questionnaire was developed based on a comprehensive review of literature and expert consultations, designed to capture three main categories of information: sociodemographic factors (e.g., maternal age, occupation, education), psychological factors (e.g., perceived stress, social support), and physiological and biological factors (e.g., pregnancy history, sleep quality, and infant feeding method).

Data were collected through four independent cross-sectional surveys conducted at distinct perinatal stages: T1 (≤ 13 weeks), T2 (14-27 weeks), T3 (28 weeks to delivery), and T4 (within 6 weeks postpartum). At each stage, a separate group of participants was recruited during their routine obstetric visits, resulting in 1,047 women at T1, 543 at T2, 421 at T3, and 424 at T4. The specific variables assessed at each stage are summarized in Table 1.

**Table 1:** Sociodemographic, Psychological, and Physiological Data Collected Across Four Stages.

**Stage**	**Sociodemographic factors**	**Psychological factors**	**Physiological and biological factors**
T1	Maternal age^*^	Life events^*^	History of deliveries^*^
	Paternal age^*^	Perceived stress	History of pregnancies^*^
	Maternal ethnicity^*^	Personality susceptibility	History of ectopic pregnancy^*^
	Current residence^*^		Sleep quality
	Maternal education level^*^		
	Paternal education level^*^		
	Maternal occupation^*^		
	Paternal occupation		
	Per capita monthly household income^*^		
	Health insurance^*^		
	Marital status^*^		
	Family support^*^		
	Relationship with husband		
	Relationship with in-laws		
	Social support		
T2	Relationship with husband	Coping strategies	Sleep quality
	Relationship with in-laws	Life events	
	Social support		
	Family support		
T3	Relationship with husband		Sleep quality
	Relationship with in-laws		
	Social support		
	Family support		
T4		Parenting stress	Baby's feeding method
		Parental efficacy	Baby's gender
		Baby's gender expectation matching	Delivery complications
		Life events	Postpartum abnormalities

^*^These variables were measured at all four stages.

Participants were eligible for inclusion if they met all of the following eligibility criteria conditions:

1) Had a current singleton pregnancy; 2) Had normal thyroid function and normal pre-pregnancy serum lipid levels; 3) Were able to correctly understand and independently complete the study questionnaire; 4) Voluntarily agreed to participate in the study.

Participants were excluded if they met any of the following conditions:

1) Had a self-reported history of depression or other mental disorders prior to pregnancy; 2) Had a history of severe physical illness or head trauma; 3) Developed serious obstetric complications and/or comorbidities during pregnancy; 4) Experienced adverse obstetric outcomes, such as miscarriage, fetal abnormalities, or stillbirth; 5) Were unable to complete the questionnaire due to physical discomfort, or withdrew from the study during follow-up; 6) Had more than 20% missing questionnaire data.

Given that this study adopted a cross-sectional design, the measurement schedule for all variables was determined based on their theoretical relevance and practical feasibility. Some variables, such as family support and negative life events, were assessed at all four stages (T1-T4) due to their continuous relevance throughout the perinatal period. In contrast, other variables were measured only at specific time points to maximize their theoretical significance and measurement validity.

For instance, perceived stress and personality susceptibility were measured only at T1, as the first trimester represents the initial phase of physiological and psychological adjustment during pregnancy. Stress perception and personality vulnerability at this stage are believed to be particularly predictive of subsequent emotional responses. Coping strategies were assessed at T2, based on previous research suggesting that the second trimester is the most stable and representative period for capturing individuals' typical coping styles. Meanwhile, variables such as parenting stress, parental efficacy, and delivery complications are specific to the postpartum context and were therefore measured exclusively at T4, when such experiences become relevant and observable. Table 2 presents detailed descriptions of each variable along with their corresponding response categories. All measurement instruments demonstrated good internal consistency in this study. The Chinese version of the EPDS has shown good reliability and validity across different stages of pregnancy and postpartum in mainland Chinese samples (40–42). The Cronbach's α coefficients were 0.901 for the Edinburgh Postnatal Depression Scale (EPDS), 0.857 for the Family Support APGAR, 0.780 for the Perceived Stress Scale (PSS), 0.900 for the Simplified Coping Style Questionnaire (SCSQ), 0.961 for the Life Events Scale for Pregnant Women (LESPW), 0.910 for the Parenting Stress Index-Short Form (PSI-SF), 0.820 for the Parental Sense of Competence Scale (PSOC), and 0.845 for the Pittsburgh Sleep Quality Index (PSQI).

**Table 2:** Description and Classification of Variables.

**Variable**	**Description**	**Response options / categories**
Maternal age	Age of the pregnant woman	18–23; 24–35; ≥36
Paternal age	Age of the partner or husband	18–24; 25–36; ≥37
Maternal ethnicity	Ethnic background of the mother	Non-ethnic minority; Ethnic minority
Current residence	Urban or rural residence	Rural Area; Town; City
Maternal education level	Mother's highest education level	Bachelor's or above; associate degree; high school; middle school or below
Paternal education level	Partner's highest education level	Bachelor's or above; associate degree; high school; middle school or below
Maternal occupation	Employment/occupation of the mother	Worker/farmer; administrative worker; service industry; professional/technical; other
Paternal occupation	Employment/occupation of the partner	Worker/farmer; administrative worker; service industry; professional/technical; other
Per capita monthly household income	Monthly household income per capita	~4000; 4001–6000; 6001–8000; ≥8001
Health insurance	Access to health insurance	New rural cooperative medical scheme; urban resident medical insurance; commercial insurance; none
Marital status	Marital status of the mother	Married; other
Relationship with husband	Perceived relationship quality with husband	Scale score
Relationship with In-laws	Perceived relationship quality with in-laws	Scale score
Family support	Support from family	Scale score
Social support	Support from social network	Scale score
Life events	Exposure to negative life events	Scale score
Sleep quality	Subjective sleep quality	Scale score
Perceived stress	Self-reported stress level	Scale score
Personality susceptibility	Dispositional traits	Scale score
Coping strategies	Cognitive/behavioral coping	Scale score
History of deliveries	Number of previous births	Yes; no
History of pregnancies	Total number of pregnancies	Yes; no
History of ectopic pregnancy	Past ectopic pregnancy	Yes; no
Baby's feeding method	Infant feeding type	Breastfeeding; formula feeding; mixed feeding
Baby's gender	Sex of the baby	Male; female
Delivery complications	Medical complications at birth	Yes; no
Postpartum abnormalities	Abnormal postpartum conditions	Yes; no
Parenting stress	Stress related to parenting	Scale score
Parental efficacy	Confidence in parenting	Scale score
Baby's gender expectation matching	Match between expected and actual gender	Matched; not matched

### 2.3 Perinatal depression

PND was assessed using the Chinese version of the Edinburgh Postnatal Depression Scale (EPDS) (43). The EPDS consists of 10 items, with each item scored from 0 to 3. The total score ranges from 0 to 30, with higher scores indicating a higher likelihood of having depression. The validity and reliability of the Chinese version of the EPDS have been reported, with a sensitivity of 0.82 and a specificity of 0.86, mirroring the diagnostic accuracy of the original scale (44). In this study, participants with an EPDS score ≥10 were classified as having PND.

### 2.4 Statistical analysis

Descriptive statistics were used to summarize the sample characteristics, with the Shapiro-Wilk test employed to assess the normality of continuous variables. Normally distributed variables are presented as mean (standard deviation), while non-normally distributed variables are reported as median (interquartile range). Categorical variables are expressed as frequencies and percentages. To identify risk factors for PND across the four periods (T1, T2, T3, and T4), univariate and multivariate logistic regression analyses were performed. The association between exposures and PND was expressed using odds ratios (OR) and 95% confidence intervals (95% CI). Variable selection was primarily guided by clinical relevance and prior evidence from the literature, ensuring that key predictors were retained in the analysis (45–48). In addition, univariate analyses (*p* < 0.10) were used as a supplementary criterion to assist in identifying potentially relevant variables that might otherwise be overlooked. This threshold was chosen to ensure that relevant correlates were not prematurely excluded while maintaining a balance between model complexity and interpretability. All selected variables were simultaneously entered into the multivariable logistic regression model to evaluate their independent effects, with statistical significance set at *p* < 0.05. To assess multicollinearity, the variance inflation factor (VIF) was calculated for all variables, and all variance inflation factors were below 5, indicating no multicollinearity among predictors (see Supplementary Table S1). All analyses were conducted using Python 3.9 and Jupyter Notebook 6.4.8.

## 3 Results

### 3.1 Descriptive statistics

**Figure 1:**
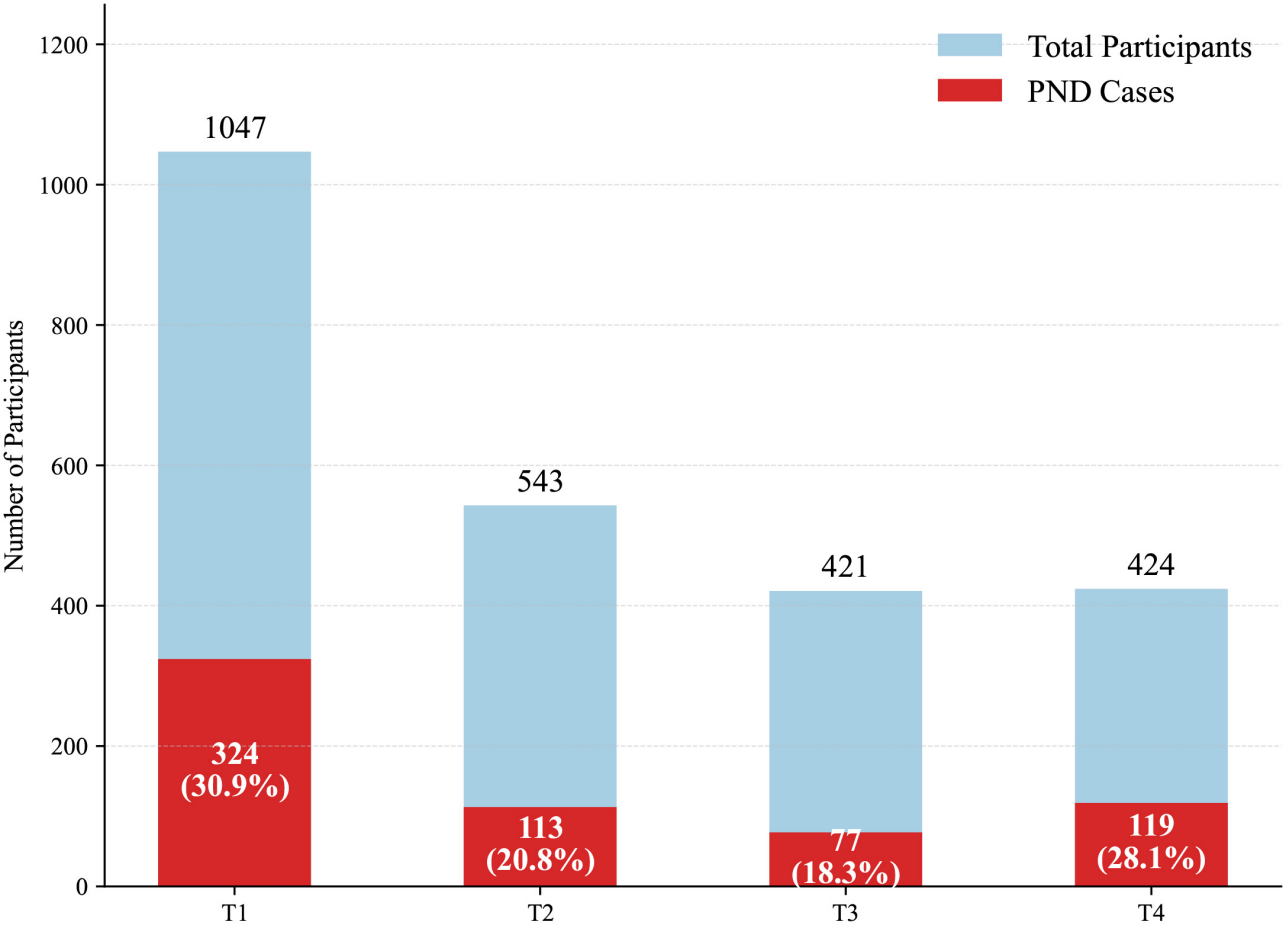
PND prevalence at each time point.

This study conducted an in-depth analysis of the prevalence of perinatal depression and its influencing factors in pregnant women at different stages of pregnancy in China. The sample size of pregnant women in the four stages of early pregnancy (T1), mid-pregnancy (T2), late pregnancy (T3) and postpartum (T4) is shown in Figure 1. The highest prevalence was observed in T1, at 30.95%. At Time Point 1, among the 1,047 participants involved in the study, a total of 324 individuals were identified as exhibiting symptoms of PND. During the second stage, 113 out of 543 participants (20.8%) were found to have symptoms of PND. In the third stage, 77 cases were observed among 421 participants (18.3%), representing the lowest prevalence across all time points. By the postpartum stage, however, the number rose again, with 119 of 424 women (28.1%) experiencing PND, marking a notable rebound compared with mid-to-late pregnancy, likely reflecting the challenges of parenting stress, physical recovery, and the transition to motherhood.

**Figure 2:**
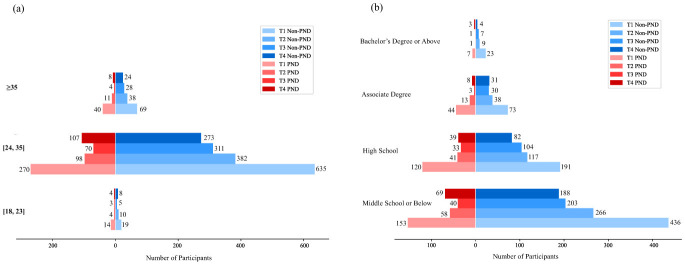
**(a)** Age distribution by PND status across pregnancy stages; **(b)** Education level distribution by PND status across pregnancy stages.

As shown in Figure 2a, most participants were aged 24-35 years, including 635 in the non-PND group and 270 in the PND group at T1, with smaller proportions in the 18 23 and ≥35 age groups. Figure 2b illustrates that the majority of participants had a high school education or below. In summary, across all stages, the study population was predominantly composed of married women aged 24–35 years with a high school education or below, who resided in urban areas and were covered by urban resident medical insurance. This demographic profile is consistent with the patient population of a tertiary hospital in a provincial capital and reflects the common characteristics of the childbearing population in similar Chinese urban settings. The complete distributions of all sociodemographic, psychological, and physiological variables for groups at each stage are provided in detail in the respective results tables for reference.

### 3.2 The prevalence and influencing factors of PND in each stage from T1 to T4

#### 3.2.1 Early pregnancy (T1)

**Figure 3:**
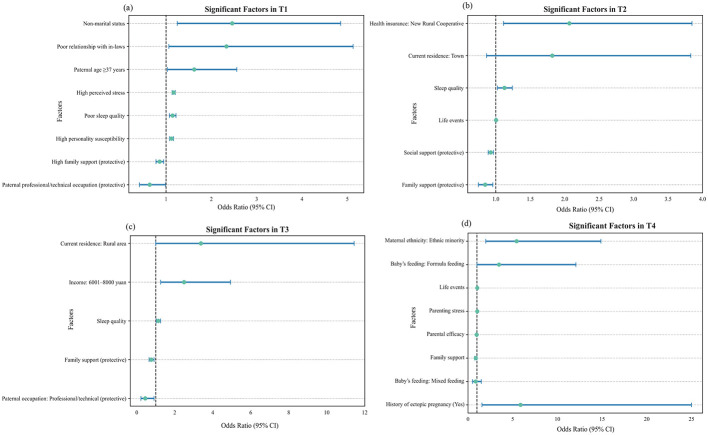
Forest plots of significant factors associated with perinatal depressive symptoms across four stages: **(a)** T1 (early pregnancy), **(b)** T2 (mid-pregnancy), **(c)** T3 (late pregnancy), and **(d)** T4 (postpartum).

At this stage, several socio-demographic and psychosocial factors were significantly associated with perinatal depressive symptoms, as shown in Figure 3a and Supplementary Table S2. Among the socio-demographic factors, paternal age over 36 years (OR = 1.62, 95% CI [1.03, 2.56]), non-marital status (OR = 2.46, 95% CI [1.25, 4.85]), and poor relationships with in-laws (OR = 2.33, 95% CI [1.06, 5.13]) were identified as risk factors, whereas paternal occupation as professional or technical personnel (OR = 0.64, 95% CI [0.41, 0.99]) and high family support (OR = 0.86, 95% CI [0.78, 0.95]) served as protective factors. In terms of psychological factors, high personality susceptibility (OR = 1.12, 95% CI [1.08, 1.17]) and elevated perceived stress (OR = 1.17, 95% CI [1.13, 1.20]) were positively associated with depressive symptoms. Additionally, within the physiological and biological domain, poor sleep quality (OR = 1.15, 95% CI [1.08, 1.22]) was also identified as a significant risk factor.

#### 3.2.2 Mid-pregnancy (T2)

At T2, among the 543 participants, 113 (20.81%) were identified as having symptoms of PND. Figure 3b and Supplementary Table S3 presents the univariate and multivariate analysis results for this stage. Within the socio-demographic domain, enrollment in the New Rural Cooperative Medical Scheme (OR = 2.07, 95% CI [1.11, 3.85]) was associated with increased risk, whereas high family support (OR = 0.85, 95% CI [0.75, 0.96]) and high social support (OR = 0.93, 95% CI [0.89, 0.97]) emerged as protective factors. Among psychological factors, negative life events (OR = 1.00, 95% CI [1.00, 1.01]) were identified as a risk factor. In terms of physiological and biological factors, poor sleep quality (OR = 1.13, 95% CI [1.02, 1.24]) was also significantly associated with PND risk.

#### 3.2.3 Late pregnancy (T3)

During late pregnancy, higher household income within a specific range and poor sleep quality were associated with an increased risk of depressive symptoms, as shown in Figure 3c and Supplementary Table S4, whereas professional paternal occupation and strong family support exerted protective effects. Specifically, a per capita monthly household income of 6,001-8,000 RMB (OR = 2.49, 95% CI [1.26, 4.94]) and poor sleep quality (OR = 1.13, 95% CI [1.03, 1.25]) were identified as risk factors, while paternal occupation as professional or technical personnel (OR = 0.45, 95% CI [0.22, 0.91]) and high family support (OR = 0.77, 95% CI [0.65, 0.91]) served as protective factors.

#### 3.2.4 Postpartum (T4)

At the postpartum stage (T4), ethnic minority status, lower family support, greater exposure to negative life events, higher parenting stress, weaker parental efficacy, a history of ectopic pregnancy, and formula feeding were significantly associated with postnatal depressive symptoms shown in Figure 3d and Supplementary Table S5. Specifically, being an ethnic minority was identified as a strong sociodemographic risk factor (OR = 5.44, 95% CI [1.99, 14.87]). Among psychosocial factors, higher life event scores (OR = 1.04, 95% CI [1.01, 1.06]) and greater parenting stress (OR = 1.03, 95% CI [1.01, 1.04]) increased the likelihood of postpartum depressive symptoms, whereas greater family support (OR = 0.87, 95% CI [0.75, 1.00]) and stronger parental efficacy (OR = 0.93, 95% CI [0.90, 0.97]) acted as protective factors. Regarding physiological and biological factors, a history of ectopic pregnancy was associated with increased odds of PND (aOR = 5.88, 95% CI [1.56, 25.00]). Formula feeding (vs breastfeeding) was also associated with marginally elevated odds of PND (aOR = 3.47, 95% CI [1.00, 12.07]).

## 4 Discussion

### 4.1 PND influencing factors

#### 4.1.1 Recurring influencing factors across multiple stages

This study identified a variety of socio-demographic, psychological, and physiological factors that influenced PND at different stages of the perinatal period. The impact of these factors varied considerably depending on the specific stage of pregnancy or postpartum, emphasizing the complexity of PND risk and the need for stage-specific interventions. In this discussion, we will first examine the factors that influenced PND across multiple time points, followed by a detailed analysis of factors that were unique to each stage.

(1) Socio-demographic factors

**High family support** consistently emerged as a significant protective factor against PND across all pregnancy stages. Early in pregnancy, it helped women manage emotional and physiological changes, while continued support reduced stress from health concerns and lifestyle adjustments in later stages. As delivery approached, strong family involvement further buffered anxiety and physical discomfort. Mechanistically, family support may protect against perinatal depression through both biological and psychosocial pathways. Biologically, greater support is associated with attenuated increases in placental corticotropin-releasing hormone (pCRH) and fewer postpartum depressive symptoms, suggesting modulation of neuroendocrine activity (49). Psychosocially, supportive relationships enhance trust, acceptance, and parenting self-efficacy (50), while practical assistance reduces fatigue and emotional burden (51). Conversely, insufficient or disrupted support may heighten emotional distress and weaken emotion regulation, consistent with digital behavioral evidence linking reduced support to increased depressive expression (52). Ultimately, cohesive and supportive family relationships foster secure attachment and maternal-infant bonding, thereby promoting resilience and protecting maternal mood (53–57).

**Paternal occupation** showed stage-dependent effects on PND risk. During pregnancy, professional and technical roles were protective, likely due to greater financial stability and flexibility, which reduce household stress and enable more emotional and practical support for expectant mothers (58).

(2)Psychological Factors

**Negative life events** emerged as a significant psychological risk factor for PND across multiple stages, particularly during T2 and T4. In T2, the occurrence of various pregnancy-related complications, such as gestational diabetes mellitus (GDM), constitutes a primary mechanism by which negative life events increase susceptibility to depressive symptoms (59), as these health issues directly heighten both the physical and psychological burden on expectant mothers. Moreover, the accumulation of multiple stressors, including personal or family conflicts and financial pressures, may exacerbate anxiety related to childbirth, further elevating the risk of depression (60). Recent findings further refine our understanding of these psychosocial mechanisms, indicating that stressful life events, personality traits, and attachment insecurity jointly predict depressive severity during pregnancy and postpartum (121).

During the postpartum period, the cumulative effects of negative life events intensify over time, and unresolved or persistent stressors may increasingly strain maternal emotional wellbeing (61–64). Concurrently, the physiological demands of recovery and the potentially traumatic experiences associated with childbirth amplify psychological burden (65). Individual characteristics further modulate this risk: mothers with higher levels of neuroticism are more prone to respond negatively to adverse life events, thereby increasing susceptibility to PND, whereas greater psychological flexibility serves as a protective factor, enabling mothers to cope more effectively with stressors and reducing the likelihood of developing depressive symptoms (66).

(3)Physiological and biological factors

**Poor sleep quality** was closely associated with PND, a finding consistently validated across multiple stages of pregnancy (64, 67–69), including the first trimester, second trimester, and third trimester. Hormonal fluctuations, fatigue, and psychological stress in early pregnancy can disrupt sleep and impair emotional regulation, while physical discomfort and anxiety in later trimesters further aggravate sleep disturbance. Biologically, changes in prolactin secretion and sustained elevations in cortisol contribute to fragmented sleep and increased arousal, reinforcing depressive vulnerability (70–72). Conversely, depressive symptoms can worsen sleep continuity through heightened arousal and neuroendocrine dysregulation, indicating a bidirectional relationship (73, 74). Prolonged sleep deprivation also induces inflammatory activation—elevated TNF-α and C-reactive protein have been observed in women with perinatal depression, particularly among those with trauma histories (75). Thus, sleep disturbance functions both as a precursor to and a consequence of perinatal depression through intertwined neuroendocrine and inflammatory pathways.

Taken together, these factors are all associated with PND across the perinatal period, but their effects varied substantially at different stages and should not be interpreted as uniform influences. Family support primarily offers emotional buffering against hormonal stress in early pregnancy (T1), while serving more instrumental roles in alleviating physical and logistical burdens during mid-to-late stages (T2-T3). The protective effect of paternal occupation also shifts, becoming a risk factor postpartum due to changes in role demands. Negative life events show peak sensitivity in T2, often linked to pregnancy complications, whereas their impact in T4 depends more on the persistence and resolution of stressors. Sleep quality was consistently associated with PND across T1-T3, with mechanisms evolving from hormonal disruption to physical discomfort and inflammatory activation, suggesting possible emotional recovery through behavioral compensation and hormonal normalization.

#### 4.1.2 Stage-specific influencing factors

(1) Early Pregnancy (T1)

The analysis of socio-demographic factors revealed that paternal age over 36, non-marital status, and poor relationship with in-laws were significant risk factors for PND. Advanced paternal age may pose challenges related to the psychological and financial expectations of parenthood. Older fathers might face increased stress due to societal pressures to provide and support a family, which can indirectly affect maternal mental health (76, 77). Additionally, the dynamics of parental age can contribute to differing views on parenting roles, potentially creating tension (76, 77). Women who are unmarried during pregnancy may face not only a lack of emotional and financial support from a partner but also significant social stigma (78–82). The cultural pressures of being unmarried while pregnant can amplify feelings of isolation, anxiety, and even shame, further increasing vulnerability to depression. Furthermore, a poor relationship with in-laws can lead to high levels of stress for pregnant women, as they may feel judged or unsupported during a critical time when family cohesion is most needed (83–85). In many cases, Chinese families live in multigenerational households or maintain close ties, and conflicts with in-laws can create a tense living environment, directly affecting maternal wellbeing. The strain of balancing cultural expectations, family roles, and personal needs in such an environment can overwhelm a mother's emotional resilience, increasing her risk of PND (83–85).

Among the psychological factors, high personality susceptibility and high perceived stress were identified as significant risk factors for PND. Women with high personality susceptibility are more prone to heightened anxiety, mood fluctuations, and emotional sensitivity, particularly during major life changes like pregnancy. The rapid physical and hormonal shifts in T1 can trigger intense emotional reactions, increasing their vulnerability to depressive symptoms (68, 78). Their pre-existing psychological vulnerability amplifies their emotional responses, placing them at greater risk for PND. High perceived stress was also strongly associated with PND risk, as women who experience high levels of stress during T1 may struggle to cope with the psychological and physiological demands of this critical period. Pregnancy is a time of substantial change, and when combined with personal, financial, or social stressors, these demands can overwhelm coping mechanisms (86–89). Chronic stress also negatively impacts sleep and physical health, further exacerbating emotional instability and increasing the likelihood of depressive symptoms (88, 89). This finding aligns with previous research indicating that anxious attachment and perceived inadequate support both predict prenatal and postnatal depression, with stress-related biological sensitization potentially serving as a mediating factor (53). Elevated TNF-α and acute-phase protein levels were observed in patients with perinatal depression and those exposed to trauma, suggesting that chronic stress and attachment insecurity may synergistically exacerbate depressive vulnerability through inflammatory pathways (75). These discoveries validate the biological mechanism through which psychosocial adversity during pregnancy may take root via immune and neuroendocrine dysregulation, thereby amplifying the impact of perceived stress on emotional well-being.

(2) Mid-pregnancy (T2)

The analysis of socio-demographic factors revealed that enrollment in the New Rural Cooperative Medical Scheme was a notable risk factor for PND during T2. Although the New Rural Cooperative Medical Scheme was designed to improve healthcare access for rural populations, it may not provide the comprehensive coverage or specialized prenatal services that pregnant women in urban areas can access. During T2, when regular prenatal checkups and early interventions are crucial, the limitations of this healthcare scheme can lead to increased anxiety and stress (90). Additionally, the lack of robust healthcare resources can hinder timely interventions for potential complications, exacerbating feelings of insecurity and vulnerability (90). In contrast, high social support emerged as a protective factor against PND. A strong social support network is crucial in easing the emotional and psychological burdens of pregnancy. Emotional and practical support from family and friends acts as a buffer against stress and anxiety, providing reassurance and security (78, 80, 91). This support helps pregnant women manage challenges more effectively, especially in settings with limited healthcare resources, and can also offer access to informal healthcare advice and community resources, bridging gaps in formal care (78, 80, 91).

(3) Late Pregnancy (T3)

The analysis of socio-demographic factors revealed that a per capita monthly household income of 6,001–8,000 RMB was a significant risk factor for PND during T3. Although this income level indicates a moderate degree of financial stability, it may still fall short in covering the additional costs associated with pregnancy and childbirth, including medical expenses, preparations for childcare, and potential loss of income during maternity leave (78, 80, 91). During the late stages of pregnancy, when families are preparing for the arrival of a new child, the financial pressures can become overwhelming. Families in this income bracket often struggle to balance everyday living costs with the heightened financial demands of pregnancy, resulting in increased stress and anxiety (78, 80, 91).

(4) Postpartum (T4)

The analysis of socio-demographic factors revealed that maternal ethnicity as an ethnic minority and current residence in a town were identified as significant risk factors for PND. One reason for the elevated risk of PND among ethnic minority women in China is that many minority groups live in remote and underdeveloped regions, where limited healthcare infrastructure and economic disparities restrict access to comprehensive maternal health services. These geographic challenges, coupled with economic disparities, heighten the barriers faced by minority mothers, increasing their susceptibility to isolation and inadequate support during T4 (77, 92–94). Additionally, cultural differences and a lack of tailored healthcare interventions further amplify the psychological stress experienced by these mothers, making them more vulnerable to depressive symptoms (77, 94). Cultural and linguistic differences can further hinder effective communication with healthcare providers (95), while the stigma surrounding mental illness may discourage professional help-seeking and instead lead women to rely on traditional or religious coping strategies (96). Evidence also shows that healthcare systems often lack cultural sensitivity, and implicit bias or systemic discrimination can result in lower referral and treatment rates among minority women, even when their psychological distress is greater (97, 98). Historical trauma and ongoing experiences of discrimination may impose chronic psychological stress, while cultural beliefs about motherhood and parenting that conflict with biomedical practices can further intensify vulnerability to depression (99). In addition, standard screening tools and interventions may lack cultural validity, underscoring the need for more culturally adapted approaches. Addressing these inequities will require not only improving service accessibility and quality, but also strengthening the cultural competence of healthcare professionals, integrating maternal mental health services into community and faith-based systems, and developing policies that explicitly support the perinatal mental health of ethnic minority women.

Among psychological factors, high parenting stress was a significant risk factor for PND. It reflects the pressures mothers face as they adapt to new responsibilities, sleep deprivation, and the physical and emotional demands of infant care, particularly during T4 (100–102). Conversely, strong parental efficacy served as a protective factor, enabling mothers to approach challenges with confidence and resilience. Believing in one's caregiving ability can mitigate stress and reduce the risk of anxiety and depressive symptoms (103–106).

Within physiological and biological factors, a history of ectopic pregnancy and formula feeding were identified as significant risk factors for PND. Previous ectopic pregnancy may cause persistent anxiety and trauma that heighten emotional distress during subsequent pregnancies (107–112). In societies where breastfeeding is idealized, mothers who use formula may experience guilt, perceived inadequacy, and social stigma, further increasing stress and isolation (113–118). Formula feeding may also lower parenting self-efficacy (PSE), negatively affecting maternal mental health (119). Therefore, healthcare providers should offer nonjudgmental, evidence-based feeding guidance and emotional support tailored to maternal needs and preferences (120).

### 4.2 Implication

These findings have substantial implications for clinical practice and public health. Early pregnancy and the postpartum period should be regarded as critical windows for screening and timely intervention for perinatal depression. The results support the integration of family-based approaches into perinatal care, as strengthening partner and family involvement can enhance emotional support, reduce maternal stress, and underscore the importance of embedding psychosocial dimensions within routine obstetric services. Moreover, the close associations between sleep disturbance, stressful life events, and PND highlight the need for multidisciplinary strategies that encompass sleep management, stress regulation, and psychological counseling. The present findings also provide a foundation for optimizing in-hospital screening protocols and promoting multidisciplinary collaboration. Strengthening cooperation among obstetric, psychiatric, and nursing teams could improve the early identification and management of high-risk women. At the policy level, these results align with the Healthy China 2030 framework, which prioritizes maternal mental health. Expanding mental health coverage, refining referral systems, and extending integrated screening models to community and primary care settings will further advance the equity and effectiveness of perinatal depression prevention and care.

### 4.3 Limitation

This study advances stage-specific risk identification of perinatal depression by systematically characterizing psychosocial and obstetric factors across pregnancy. However, several limitations should be acknowledged. We relied on the EPDS with a cutoff of ≥ 10 as a screening tool without clinical diagnostic interviews, which may have inflated scores and obscured gradient effects. This threshold has been widely adopted in Chinese perinatal studies to balance sensitivity and specificity for screening purposes; however, it identifies a broader range of women with elevated depressive symptoms rather than only those meeting diagnostic criteria for major depression. Consequently, the prevalence estimates in this study likely reflect the presence of possible depressive symptoms rather than clinically confirmed cases, and some degree of score inflation cannot be ruled out. Nevertheless, the use of a lower cutoff enhances sensitivity for detecting women at risk who might otherwise remain unrecognized in routine care, which aligns with the preventive focus of perinatal mental health screening. Therefore, the reported prevalence represents depressive symptom levels rather than clinically confirmed depression, and comparisons across studies should consider differences in EPDS cutoff values.

The single-center sampling design may limit the generalizability of the findings. As is well recognized, participants were recruited from a tertiary hospital located in an urban area, and women attending such centers often have higher socioeconomic status, better health awareness, and greater access to medical resources than those in community or rural settings. Therefore, our findings may be more applicable to women seeking care in similar high-level medical centers rather than directly generalizable to those in primary care or community settings. Hence, caution is warranted when extrapolating the observed prevalence, risk factors, or associations to broader populations. To enhance generalizability, future research should be conducted across multiple centers, different levels of healthcare institutions, and community populations to validate these findings and strengthen their external validity.

In addition, the use of self-reported measures may introduce recall or reporting bias. Our analyses reveal associations between different perinatal stages rather than causal or temporal relationships. Longitudinal studies are still needed to determine whether these associations have predictive value and to clarify their potential causal pathways.

## 5 Conclusion

This study demonstrates that perinatal depression (PND) varies dynamically across stages, with heightened risk during early pregnancy and postpartum. By identifying both stable and stage-specific predictors, it advances understanding of how psychosocial, physiological, and biological factors interact over time to shape maternal mental health. These findings enrich the life-course perspective of PND, emphasizing that risk mechanisms are not static but evolve in timing and intensity. Recognizing such stage-dependent patterns provides a foundation for more targeted, evidence-based screening and intervention strategies, and for future research exploring the dynamic biopsychosocial pathways underlying perinatal mood disorders.

## Data Availability

The raw data supporting the conclusions of this article will be made available by the authors, without undue reservation.
